# Effects of Olive Leaf Extracts as Natural Preservative on Retailed Poultry Meat Quality

**DOI:** 10.3390/foods9081017

**Published:** 2020-07-29

**Authors:** Ebeed Saleh, Alaa Eldin Morshdy, Eman El-Manakhly, Sarah Al-Rashed, Helal F. Hetta, Philippe Jeandet, Ramadan Yahia, Gaber El-Saber Batiha, Eman Ali

**Affiliations:** 1 Food Hygiene and Control Department, Faculty of Veterinary Medicine, Damanhour University, Damanhour 22511, El-Beheira, Egypt; eman08574@gmail.com; 2 Food Hygiene and Control Department, Faculty of Veterinary Medicine, Zagazig University, Sharkia 44511, Egypt; alaaelmorshdy1952@gmail.com; 3 Department of Botany and Microbiology, College of Science, King Saud University, Riyadh 11451, Saudi Arabia; salrashed@ksu.edu.sa; 4 Department of Medical Microbiology and Immunology, Faculty of Medicine, Assiut University, Assiut 71515, Egypt; hettahf@ucmail.uc.edu; 5 Department of Internal Medicine, University of Cincinnati College of Medicine, Cincinnati, OH 45221, USA; 6 Research Unit “Induced Resistance and Plant Bioprotection”, EA 4707, SFR Condorcet FR CNRS 3417, Faculty of Sciences, University of Reims, PO Box 1039, 51687 Reims CEDEX 2, France; philippe.jeandet@univreims.fr; 7 Department of Microbiology and Immunology, Faculty of pharmacy, Deraya University, Minia 61512, Egypt; ramadanfarrag@rocketmail.com; 8 Department of Pharmacology and Therapeutics, Faculty of Veterinary Medicine, Damanhour University, Damanhour 22511, AlBeheira, Egypt

**Keywords:** poultry meat, olive leaf extract, microbial quality, natural preservative

## Abstract

Poultry meat is commonly marketed at refrigerated temperatures (2–5 °C). The major concern for retailers and consumers is the quality and safety of refrigerated poultry meat. During the chilling period, poultry meat undergoes too many undesirable changes due to microbial growth that leads to spoilage and economic loss. Therefore, this study was conducted to assess the effects of olive leaf extracts (OLE) used at three concentrations (0.25, 0.5, and 1%) on the sensory attributes, as well as the chemical and microbiological quality of raw poultry meat stored at 4 ± 1 °C for 15 days. The results revealed that the OLE addition reduced microbial growth successfully, and maintained the chemical quality and sensory attributes of poultry meat. Moreover, OLE extended the shelf-life of the poultry meat that held under proper refrigeration conditions up to 15 days compared to the control group, that was completely spoiled by the sixth day of storage. This study concludes that OLE could be used both as a natural antioxidant and an antimicrobial preservative for chilled poultry meat held at refrigerated temperature.

## 1. Introduction

Chicken meat is a good protein source with high biological value containing thiamine, nicotinic acid, vitamin A, iron, and phosphorus [1]. Additionally, chicken meat’s low energy value places it as a nutritious food suggested for use in healthy diets. Poultry meat has less fat content, and a higher ratio of monounsaturated and polyunsaturated fatty acids when compared to other meat species [2]. It is well known that poultry meat is liable to rapid deterioration when kept under inadequate storage conditions. Consequently, poultry meat is often the cause of alimentary infections. Since poultry meat is not consumed raw, epidemics develop as the result of a secondary contamination during production, storage or preparation [2]. Poultry microflora is transferred from the primary production sites to production lines, and even further, by subsequent contaminations [2]. It is known that poultry is a reservoir for many bacteria that may be pathogenic to humans. Low levels of sanitation may pose a threat to the consumer if the product is not treated in a safe manner [3]. The bacterial contamination and hygienic status during the meat production process could be measured using the aerobic plate count and total Enterobacteriaceae [4]. It is well known that olive leaf extracts (OLE) are considered as a natural source of phytochemical compounds, especially phenolics [5,6]. OLE might be used as a natural source of phytochemicals including tyrosol, oleuropein, phenolic acids, hydroxytyrosol, apigenin 7-*O*-glucoside, rutin, luteolin 4-*O*-glucoside, and caffeic acid [7,8]. Phenolic compounds could inhibit the growth, proliferation and enterotoxin production of *Staphylococcus aureus* [9]. Polyphenolic compounds from OLE act as free radical scavengers breaking the free radical chain reaction [10]. The bioactivation of these compounds may also contribute to their antioxidant activity through the inhibition of metal ion chelation [11,12]. Olive leaves have already been used in chicken diet to improve the oxidative stability, physicochemical and sensory properties of chicken meat during freezing [13]. In addition, OLE has been shown to enhance the quality and shelf life of meat products [14]. In relation to the above, the aim of this study was to examine the shelf life of chilled poultry meat in Egypt after the addition of different concentrations of olive leaf extracts and its effect on both the physicochemical properties and the microbial quality of poultry meat.

## 2. Materials and Methods

### 2.1. Extraction of Olive Leaf Extracts

Olive leaf parts were washed using distilled water, then dried on an oven at 50 °C and ground to a fine powder using a blender [15,16]. We used 100% absolute ethanol as an organic solvent for extraction. The extraction was done using a Soxhlet apparatus (Garg Process Glass India Private Limited/India). The sample (25 g of olive leaves) was placed in thimble-holder using filter paper inside the main chamber of the apparatus, which was gradually filled with condensed fresh extraction solvent (200 mL) from a distillation flask. When the solvent reaches the overflow level, a siphon aspirates the solutes from the thimble-holder and returns them to the distillation flask. Sample extraction in the Soxhlet apparatus took 10–12 h at 40–80 °C. The solvent was removed under rotatory evaporation, yielding the extracted compounds [17]. A non-soluble portion of the plant sample remained in the thimble and was usually discarded [18].

### 2.2. Preparation of Poultry Meat Samples

A total of 96 chilled poultry meat slices from chicken breast (100 ± 10 g), 2 cm in thickness, were purchased from butchers in Damanhour city, El-Behera governorate, Egypt. The samples were rapidly transferred in separate sterile and labeled plastic bags in an icebox under complete aseptic conditions without undue delay [19]. The poultry meat samples were divided into two groups; treated and control ones. The treated groups were subdivided into 3 groups (18 samples in each group) that were dipped for 15 min in OLE at concentrations of 0.25% (T1), 0.5% (T2), and 1% (T3) (0.25, 0.5, and 1 g of pure OLE to a final volume of 100 mL of sterile distilled water, respectively). The samples were drained well for 5 min on a sterile stainless wire mesh screen. The treated and control samples were stored at 4 ± 1 °C and examined regularly every 3 days at (0, 3rd, 6th, 9th, 12th, and 15th) for chemical, microbiological and sensory parameters. The scheme was replicated 4 times/day every 3 days for 15 days of storage.

### 2.3. Sensory Evaluation

Twenty panelists (adults, males above 40 years, untrained), each one served chicken breast (100 ± 10 g) for each concentration, were asked to assess the sensory qualities of each sample. The samples were blind coded with a specific number; the panelists were not informed about the experimental approach [20,21,22]. They were asked to give a score for each of overall acceptance (color, odor and texture) while the samples were fresh (uncooked). Then, the samples without salt and spices were cooked and attended to the panelists to complete the evaluation of the sensory qualities. The panelists were asked to drink warm water between the samples. A nine-point descriptive scale was used. A score of 7–9 indicated ‘‘very good’’ quality, a score of 4.0–6.9 indicated ‘‘good’’ quality, a score of 1.0–3.9 indicated “spoiled”. This was used for the evaluation of the appearance, smell, texture, taste, and the overall acceptability. Sensory evaluation was repeated after each treatment.

### 2.4. Chemical Analysis of Treated Meat Samples

- pH measurement [23] was verified using a Jenco pH handheld meter-609. pH calibration was done via knobs and pH slope calibration potentiometer with calibration standards (EOS 63-11/2006). Ten grams of the examined chicken fillet samples were homogenized with 25 mL neutral distilled water, left to stand for 10 min, then filtered. The calibration of the pH meter was done using buffer solutions of exactly known pH standards (pH 7.01 and 4.01).
- The total volatile basic nitrogen content (TVBN) (mg/100 g) was analyzed according to the method recommended by [23] (EOS 63/10- 2006). Ten grams of chicken fillets sample were minced in a chopper for 1–2 min till homogenization. In a distillation flask, 2 g of magnesium oxide were added. Distilled water (300 mL) was added to the minced sample. The distillation was performed and 100 mL distillate was received within 30 min in a beaker containing 25 mL 2% boric acid. Then, titration against H_2_SO_4_ 0.1 M was performed until a faint pink color was obtained. Calculation: The amount of TVBN was calculated from the volume of 0.1 M sulfuric acid used for titration and calculated as: TVBN mg/100 g = R × 14, where R is the volume of H_2_SO_4_ exhausted in titration.
- The determination of thiobarbituric acid (TBA) [23]: the TBA number was conveyed as milligrams of malondialdehyde equivalents per kilogram of samples (EOS 63/9-2006). Ten grams of the sample were blended with 48 mL distilled water. Two ml of 4% ammonium chloride (to bring the pH to 1.5) was added to the previous contents in a warring blender for 2 min and left at room temperature for 10 min. The mixture was quantitatively transferred into Kjeldal flasks by washing with an additional 50 mL distilled water, followed by an antifoaming preparation and a few glass beads. The Kjeldal distillation apparatus was assembled and the flask was heated to 50 °C. Distillates were collected at 10 min from the time of the boiling commencing. The distillates (50 mL) were mixed, and then were pipette into a glass Stoppard tube. Then, 5 mL TBA reagent (0.2883/100 mL of glacial acetic acid) was added, the tube was stoppered, shacked and immersed in a boiling water bath for 35 min. A blank was similarly prepared using 5 mL distilled water with 5 mL of TBA reagent and treated like the sample. After heating, the tube was cooled under tap water for 10 min. A portion was transferred to a curette and the optical density (D) of the sample was read against the blank by means of a spectrophotometer (Perkin Elmer, 2380, USA) at a wave length of 538 nm. **Calculation:** the TBA value (mg malondialdehyde/Kg of sample) = Dx7.8 D: the read of sample against blank.

### 2.5. Microbiological Examination

Ten grams of the treated poultry meat sample was aseptically homogenized with 90 mL sterile peptone water 0.1% at 4000 rpm for 2.5 min. Ten-fold serial dilutions were prepared. The total aerobic bacterial count (TBC) was estimated using the plate count agar after incubation for 48 ± 2 h at 37 °C [24] Psychrophilic bacterial count (PBC) was demonstrated using the pour plate technique after incubation at 7 °C for 10 days [25]. Enterobacteriaceae count [26] was estimated using violet red bile glucose agar medium with incubation at 37 °C for 24 h. The staphylococcal count [27] was carried out using the Baird Parker agar medium with incubation at 37 °C for 48 h. The total mold and yeast count was estimated using Sabouraud’s dextrose agar (SDA) with incubation at 22–25 °C, for 5 days up to 7 days, according to [28].

### 2.6. Statistical Analysis

Data were statistically analyzed using the Statistical Analysis System (SAS, Cary, NC, USA, version 9.3) software. The chemical, microbiological, and organoleptic parameters were presented as the mean ± standard deviation “SD”. Significant means were compared using Tukey’s Studentized Range (HSD) post-hoc test (*p* ≤ 0.05) with a nested procedure model (*p* ≤ 0.05).

## 3. Results and Discussion

### 3.1. Sensory Evaluation and Overall Acceptance

The results of the overall acceptance of the poultry meat samples stored at 4 ± 1 °C revealed that the control samples were completely spoiled by the sixth day of storage. The addition of OLE at 0.25, 0.5, and 1% significantly maintained the overall acceptability of the sensory properties. OLE at 0.25% maintained the overall acceptability of the sensory properties until the ninth day, while 0.5% of OLE maintained the overall acceptability of the sensory properties until the 12th day. OLE at 1% maintained the overall acceptability of the sensory properties until the 15th day. In addition, the samples containing 1% OLE showed the highest acceptability enhancement, whereas 0.25% OLE-treated samples exhibited the least enhancement (Table 1). Our findings were in agreement with Sasse et al. [29], who reported that many herbs and spices improved both the color and flavor stability in meat. Our findings showed that OLE maintains the overall acceptability of treated poultry meat, confirming the data obtained by Shalaby et al. [30], which reported that using OLE as a natural preservative on minced beef enhanced its quality attributes including sensory attributes, as well as increased the minced beef’s shelf-life under cold storage conditions.

### 3.2. Chemical Analysis of Treated Poultry Meat

#### 3.2.1. Hydrogen Ion Concentration (pH)

The obtained results summarized in Table 2 showed that the samples treated with OLE had lower pH values than the control samples for the different time periods of analysis. In addition, by increasing the concentration of OLE to 1%, the pH values scored the highest effects in lowering the pH values for 15 days in chilled storage. Our findings revealed that lower pH values in treated poultry meat were obtained with OLE as compared to the control samples, and this may be linked to the active compounds present in olives’ leaf extract. Our observations are supported by the findings of Shalaby et al. [30], who reported lower pH values in minced meat treated with OLE as a consequence of the nature and the properties of the active phenolic compounds present in OLE. These results thus indicate that the application of OLE as an antimicrobial agent influences the pH of poultry meat during the refrigeration storage. The addition of OLE could decrease the pH values, mentioned which makes the media undesirable for the growth of most bacterial population present on the meat surface.

#### 3.2.2. Total Volatile Nitrogen (TVN)

Total volatile basic nitrogen (TVBN mg/100 g) is used as an index of raw meat quality. The results mentioned in Table 3 show that the mean values of TVN in the control samples were 3.23 ± 0.06, 18.96 ± 0.13 and 30.21 ± 0.41 mg/100 g at day 0, 3, and 6 of storage, respectively. This increase in TVN values in the meat might be attributed to the breakdown of protein, because of the activity of different microorganisms and their proteolytic enzymes [31]. In treated poultry meat, with OLE at different concentrations of 0.25, 0.5 and 1%, the TVN values changed from 3.18 ± 0.05, 3.12 ± 0.01 and 3.07 ± 0.02 mg/100 mg, respectively, at day 0 to 28.86 ± 0.44, 22.85 ± 0.23 and 19.83 ± 0.15 mg/100 mg at day 15 of storage at 4 °C, respectively. In addition, increasing the OLE concentration to 1% was more effective at reducing the TVN values than the lower concentration of OLE (0.25%) at day 15 of storage. There were significant differences between the negative control group and the other treated groups at (*p* < 0.05). According to the permissible limits established by Amin et al., [32] which stated that TVN should not exceed 20 mg/100 g, the control group exceeds the permissible limit by the sixth day of storage and becomes unfit compared to the poultry meat treated with 0.25%, 0.5 and 1% which becomes unfit after 9, 12 and 15 days of storage at 4 °C, respectively. Our findings revealed that olive leaf extracts could reduce protein decomposition and decrease the TVN values at day 15 of storage. This result is supported by Marangoni et al. [13] who reported that the use of 5 g of olive leaves per kg of feed reduced protein oxidation, leading to changes in the cell structure of myofibrillar proteins in chicken meat. The increase in the TVBN values in meat during storage might be attributed to protein breakdown as a result of microbial and proteolytic activities [33]. TVBN increases to critical values indicate the incipient spoilage of the chicken meat product samples after different periods of storage [34].

#### 3.2.3. Thiobarbituric Acid Reactive Substances (TBARs)

The thiobarbituric acid reactive substance (TBARS) assay is one of the most frequently used techniques for the measurement of secondary oxidation products, mainly malondialdehyde (MDA), regarded as the cause of oxidative rancidity, and which may contribute to the off-flavor of oxidized fat [22]. The recorded data in Table 4 reveal that the mean values of TBA in control samples were 0.06 ± 0.01, 0.81 ± 0.02, and 1.09 ± 0.05 mg MDA/kg at 0, 3 and 6 days of storage, respectively. Poultry meat treated with OLE at a 0.25% concentration sees their TBA values increasing from 0.055 ± 0.01 mg MDA/kg at day 0 of storage to 1.22 ± 0.06 mg MDA/kg at day 15 of storage. TBA values of 0.5% OLE samples increased from 0.05 ± 0.01 mg MDA/kg at day 0 of storage to 1.01 ± 0.03 mg MDA/kg at day 15 of storage. Finally, the poultry meat treated with 1% OLE have their TBA values increased from 0.045 ± 0.01 mg MDA/kg at day 0 of storage to 0.88 ± 0.01 mg MDA/kg at day 15. Therefore, the samples treated with different concentrations of OLE saw their TBA values decreasing, especially at the 9th, 12th and 15th days of storage, as compared to the control samples. Rancid flavor in the control samples started at the sixth day of storage, while the samples treated with OLE still had a normal flavor until the end of the storage period, without developing any rancidity as observed by sensory evaluation (smell). These results come in agreement with Marangoni et al. [13] who reported that chicken meat receiving OLE displayed a significant reduction in the rancid odor and flavor during the 120 days of analysis. Our findings revealed that OLE had inhibitory effects on lipid oxidation. McDonald et al. [35] have reported that there are a number of phenolic compounds in crude OLE (e.g., hydroxytyrosol and oleuropein). The inhibition of lipid oxidation and the ability to donate hydrogen is believed to be improved with the increasing amount of hydroxyl groups within polyphenolics. The antioxidant compounds present in olive leaves can increase the shelf-life of food products by delaying the lipid peroxidation process [36], hence the OLE was investigated as an additive supplement to improve the quality and stability of meat products [37].

### 3.3. Microbiological Examination of Treated Poultry Meat with OLE

#### 3.3.1. Total Aerobic Bacterial Count

The aerobic bacterial count is a commonly recognized indicator of the general level of microbial contamination in meat and its products. Alberle et al. [38] reported that meat is generally considered of poor hygienic quality or unfit for consumption when the aerobic plate count (APC) exceeds 10^6^ cfu/g. Am et al. [32] stated that the total bacterial count of chilled poultry should not exceed 10^5^/g. The data presented in Table 5 indicate that the samples treated with OLE displayed a decreasing count of aerobic plate microorganisms. Our results showed that OLE had a positive impact in decreasing the total aerobic plate count in the treated samples compared with the control one. This result was confirmed by Shalaby et al. [30] who reported that OLE has a good effect on the antimicrobial activity such as the total bacterial count in minced beef. The partial hydrophobic nature of phenolic compounds may degrade the cell wall, disrupt the cytoplasmic membrane, damage membrane proteins, and interfere with membrane-integrated enzymes, which may eventually lead to bacterial cell death [39] explaining the decrease in aerobic bacterial count. However, OLE effectively enhances the microbial counts in poultry meat samples for 9, 12 and 15 days. The OLE at 1% was more effective in decreasing the APC counts than the lowest concentrations (*p* < 0.05). Significant differences were observed between the treated samples with 0.25, 0.5, and 1% of OLE and the control samples during the storage period. These results are supported by Aytul, [40] who reported that the use of 2% and 3% OLE could affect the microbial load, the total viable and coliform counts.

#### 3.3.2. Total Psychrotrophic Count (TPC)

Results obtained in Table 6 show that the psychrophilic count of control poultry meat samples were 4.23 ± 0.10, 4.64 ± 0.06 and 5.19 ± 0.18 cfu/g after 0, 3 and 6 days of storage period, respectively. The mean of the total psychrophilic count in the treated poultry meat samples at different concentrations of OLE (0.25, 0.5 and 1%) were 4.01 ± 0.11, 3.86 ± 0.15 and 3.70 ± 0.19 log10 cfu/g), respectively, at day 0 with significant differences with control samples. Control poultry meat samples started to decompose on the sixth day, while the treated samples with 0.25 and 0.5% of OLE started to decompose at the ninth and 12th days of storage, respectively. Poultry meat samples treated with 1% OLE did not decompose until the 15th day of storage. The reduction in psychrophilic bacterial numbers correlated with the presence of active ingredients in the olive leaves such as oleuropein, tyrosol and hydroxytyrosol, which act as antimicrobial agents. These results are in agreement with Cardoso et al. [41]. Our results showed that OLE had positive impact in decreasing the psychrophilic count in the treated samples as compared to the control ones; this result was supported by Shalaby et al. [30].

#### 3.3.3. Total Enterobacteriaceae Count (TEC)

The results presented in Table 7 show that the log mean value of Enterobacteriaceae count in control samples were 3.02 ± 0.06, 3.48 ± 0.07 and 3.81 ± 0.04 cfu/g after 0, 3, and 6 days of storage, respectively. The means of the total Enterobacteriaceae counts in the treated poultry meat samples treated with different concentrations of OLE (0.25, 0.5 and 1%) were 2.92 ± 0.03, 2.82 ± 0.04 and 2.56 ± 0.07 log10 cfu/g), respectively, at day zero, with significant differences compared to the control. Control poultry meat samples started to decompose at day 6, while decomposition within the treated samples with 0.25 and 0.5% of OLE was delayed at the 9th and 12th days of storage, respectively. Treated poultry meat samples with 10% OLE did not decompose until the 15th day of storage. The obtained results highlight the positive preservative effects of OLE treatments on poultry meat samples, where it decreased the TEC compared with the control. The OLE also prolonged the shelf life of chilled poultry meat to the ninth, 12th and 15th days of storage. Our findings revealed that the OLE significantly decreased the total Enterobacteriaceae count in the treated poultry meat. These results are supported by Saadony et al. [42] who reported that the olive leaf extracts have inhibitory effects against Gram-negative bacteria, especially those from the Enterobacteriaceae family, including *E. coli* and *Salmonella*.

#### 3.3.4. Total Staphylococcal Count (TSC)

The recorded results in Table 8 show that the staphylococcal count of the control samples was 4.34 ± 0.08, 4.88 ± 0.06, and 5.19 ± 0.04 log10 cfu/g after 0, 3, and 6 days of storage, respectively. The means of the total staphylococcal counts in the treated poultry meat samples at different OLE concentrations (0.25, 0.5 and 1%) were 4.21 ± 0.01, 4.07 ± 0.07 and 3.76 ± 0.11 log10 cfu/g), respectively, at day 0 with significant differences with the control samples. Control poultry meat samples started to decompose at day 6, while the treated samples with 0.25 and 0.5% OLE started to decompose at the ninth and 12th days of storage, respectively. The poultry meat samples treated with 1% OLE did not decompose until the 15th day of storage. Samples treated with different concentrations of OLE thus showed a decreasing TSC compared with the controls, with a specific emphasis after the 12th and 15th days of storage. In addition, the OLE at 1% was more effective in decreasing the TSC counts than at 0.25%. Our findings revealed that the OLE had an important role in decreasing the count of Staphylococci. This results comes in agreement with Aliabadi et al. [43] who reported that olive leaves would have a positive impact in regulating microbial infections such as those caused by *Staphylococcus aureus*. Owen et al. [44] reported that the phenolic compounds present in OLE indeed exhibited antimicrobial activity toward various microorganisms, especially *Staphylococcus aureus*. Moreover, Markin et al. [45] documented that water olive leaf extracts at 0.6% (w/v) concentration killed *S. aureus* within three hours of exposure. Saadony et al. [42] also reported that olive leaf extract extracts were found to be effective against some pathogenic bacteria, such as *E. coli*, *Staph. aureus* and *Salmonella typhimurium*, although being more effective against Gram-positive than Gram-negative bacteria.

#### 3.3.5. Total Mold and Yeast Count

The results displayed in Table 9 show that the mold and yeast count of the control samples were 3.77 ± 0.07, 4.42 ± 0.13, and 4.72 ± 0.10 log10 cfu/g after 0, 3, and 6 days of storage, respectively. The means of total mold and yeast counts in the treated poultry meat samples at different concentrations of OLE (0.25, 0.5 and 1%) were 3.66 ± 0.05, 3.51 ± 0.07 and 3.10 ± 0.17 log10 cfu/g), respectively at day 0 with significant differences compared with the control samples. The control poultry meat samples started to decompose on the 6th day, while samples treated with 0.25 and 0.5% OLE started to decompose on the 9th and 12th days of storage, respectively. Poultry meat samples treated with 1% OLE did not decompose until the 15th day of storage. Shalaby et al. [30] have also reported that OLE has a positive effect in decreasing the total yeast and mold counts in minced beef treated with OLE. Our findings showed that the samples treated with different concentrations of OLE display decreasing total mold and yeast counts compared with the control ones with a specific emphasis after the 12th and 15th days of storage. In addition, the OLE at 1% was more effective in decreasing the total mold and yeast counts than the 0.25 and 0.5% extracts. These results come in agreement with those of Lafka et al. [46], who reported that OLE contains compounds with potent antimicrobial activity against bacteria and fungi. Moreover, Özcan et al. [47] demonstrated that OLE can act as a potent antifungal agent especially against *C. albicans*.

## 4. Conclusion

Olive leaf extract has been shown to maintain the sensory attributes as well as the chemical and microbiological quality in chilled poultry meat. OLE is a potent source of polyphenols having antioxidant and antimicrobial properties capable of inhibiting microbial growth and increasing the shelf life of poultry meat. The obtained results were concentration-dependent (the 0.25%-treated samples had a shorter shelf life when compared with the 0.5 and 1%-treated samples during the storage period), with increasing OLE concentration, leading to an enhancement of the safety and quality of the poultry meat that reduces the economic losses caused by meat decomposition.

## Figures and Tables

**Table 1:** Pattern of the overall acceptance of the fresh poultry meat treated with different concentrations of olive leaf extract during the chilling storage period at 4 ± 1 °C (mean ± standard deviation “SD”).

Storage Period	Control	Olive Leaf Extract Concentrations		
		0.25%	0.5%	1%
	Mean ± SD	Mean ± SD	Mean ± SD	Mean ± SD
Zero day	9.00 ± 0.00 ^Aa^	9.00 ± 0.00 ^Aa^	9.00 ± 0.00 ^Aa^	8.55 ± 0.25 ^Ba^
3rd day	6.70 ± 0.20 ^Db^	8.40± 0.20 ^Bb^	8.85 ± 0.15 ^Aa^	8.37 ± 0.22 ^Ca^
6th day	2.32 ± 0.12 ^Dc^	6.90 ± 0.30 ^Cc^	8.45 ± 0.15 ^Ab^	7.63 ± 0.18 ^Bb^
9th day	Decomposed	3.00 ± 0.40 ^Cd^	7.75 ± 0.11 ^Ca^	7.13 ± 0.36 ^Bc^
12th day	Decomposed	Decomposed	4.72 ± 0.46 ^Ad^	5.19 ± 0.09 ^Bd^
15th day	Decomposed	Decomposed	Decomposed	3.40 ± 0.16 ^Ae^

Means carrying a different superscript capital, with a small letter on the same row, in columns, respectively, are significantly different (*p* < 0.05).

**Table 2:** Pattern of the pH values of the poultry meat treated with different concentrations of olive leaf extract during the chilling storage period at 4 ± 1 °C (mean ± standard deviation “SD”).

Storage Period	Control	Olive Leaf Extract Concentrations		
		0.25%	0.5%	1%
	Mean ± SD	Mean ± SD	Mean ± SD	Mean ± SD
Zero day	5.71 ± 0.02 ^Ac^	5.68 ± 0.01 ^Bf^	5.67 ± 0.01 ^Bf^	5.64 ± 0.06 ^Bf^
3rd day	6.12 ± 0.04 ^Ab^	5.80 ± 0.03 ^Be^	5.76 ± 0.01 ^Bc^	5.72 ± 0.02 ^Ce^
6th day	6.44 ± 0.05 ^Aa^	6.0 ± 0.04 ^Bd^	5.91 ± 0.03 ^Cd^	5.85 ± 0.01 ^Dd^
9th day	Decomposed	6.16 ± 0.04 ^Ac^	6.06 ± 0.02 ^Bc^	6.01 ± 0.03 ^Cc^
12th day	Decomposed	6.35 ± 0.03 ^Ab^	6.23 ± 0.03 ^Bb^	6.11 ± 0.04 ^Cb^
15th day	Decomposed	6.49 ± 0.04 ^Aa^	6.36 ± 0.04 ^Ba^	6.19 ± 0.03 ^Ca^

Means carrying a different superscript capital, with a small letter on the same row, in columns, respectively, are significantly different (*p* < 0.05).

**Table 3:** Pattern of the total volatile nitrogen (TVN) (mg%) of the poultry meat treated with different concentrations of olive leaf extracts during the chilling storage period at 4 ± 1 °C (mean ± standard deviation “SD”).

Storage Period	Control	Olive Leaf Extract Concentrations		
		0.25%	0.5%	1%
	Mean ± SD	Mean ± SD	Mean ± SD	Mean ± SD
Zero day	3.23 ± 0.06 ^Ac^	3.18 ± 0.05 ^Af^	3.12 ± 0.01 ^Af^	3.07 ± 0.02 ^Af^
3rd day	18.96 ± 0.13 ^Ab^	9.06 ± 0.06 ^Be^	8.48 ± 0.07 ^Be^	8.20 ± 0.06 ^Be^
6th day	30.21 ± 0.41 ^Aa^	13.79 ± 0.06 ^Bd^	11.66 ± 0.17 ^Cd^	10.95 ± 0.09 ^Cd^
9th day	Decomposed	18.82 ± 0.16 ^Ac^	16.24 ± 0.17 ^Bc^	15.46 ± 0.13 ^Bc^
12th day	Decomposed	24.49 ± 0.11 ^Ab^	19.87 ± 0.13 ^Bb^	17.12 ± 0.14 ^Cb^
15th day	Decomposed	28.86 ± 0.44 ^Aa^	22.85 ± 0.23 ^Ba^	19.83 ± 0.15 ^Ca^

Means carrying a different superscript capital, with small letter on the same row, in columns, respectively, are significantly different (*p* < 0.05).

**Table 4:** Pattern of thiobarbituric acid (TBA) (mg/kg) of the poultry meat treated with different concentrations of olive leaf extracts during the chilling storage period at 4 ± 1 °C (mean ± standard deviation “SD”).

Storage Period	Control	Olive Leaf Extract Concentrations		
		0.25%	0.5%	1%
	Mean ± SD	Mean ± SD	Mean ± SD	Mean ± SD
Zero day	0.06 ± 0.01 ^Ac^	0.055 ± 0.01 ^Af^	0.05 ± 0.01 ^Af^	0.045 ± 0.01 ^Af^
3rd day	0.81 ± 0.02 ^Ab^	0.39 ± 0.02 ^Be^	0.33 ± 0.01 ^Ce^	0.275 ± 0.01 ^De^
6th day	1.09 ± 0.05 ^Aa^	0.66 ± 0.04 ^Bb^	0.55 ± 0.03 ^Cd^	0.49 ± 0.03 ^Dd^
9th day	Decomposed	0.84 ± 0.03 ^Ac^	0.69 ± 0.01 ^Bc^	0.615 ± 0.02 ^Cc^
12th day	Decomposed	1.02 ± 0.04 ^Ab^	0.87 ± 0.01 ^Bb^	0.745 ± 0.02 ^Cb^
15th day	Decomposed	1.22 ± 0.06 ^Aa^	1.01 ± 0.03 ^Ba^	0.88 ± 0.01 ^Ca^

Means carrying a different superscript capital, with a small letter on the same row, in columns, respectively, are significantly different (*p* < 0.05).

**Table 5:** Pattern of the aerobic bacterial count (log10 cfu/g) in poultry meat treated with different concentrations of olive leaf extract during the chilling storage period at 4 ± 1 °C (mean ± standard deviation “SD”).

Storage Period	Control	Olive Leaf Extract Concentrations		
		0.25%	0.5%	1%
	Mean ± SD	Mean ± SD	Mean ± SD	Mean ± SD
Zero day	4.25 ± 0.05 ^Ac^	4.08 ± 0.11 ^Bd^	3.93 ± 0.14 ^Cd^	3.85 ± 0.13 ^Ce^
3rd day	4.65 ± 0.02 ^Ab^	4.32 ± 0.03 ^Bc^	4.17 ± 0.02 ^Cc^	3.99 ± 0.04 ^Dd^
6th day	5.22 ± 0.06 ^Aa^	4.53 ± 0.03 ^Bb^	4.27 ± 0.03 ^Cc^	4.05 ± 0.05 ^Dd^
9th day	Decomposed	4.71 ± 0.02 ^Aa^	4.48 ± 0.06 ^Bb^	4.20 ± 0.09 ^Cc^
12th day	Decomposed	Decomposed	4.67 ± 0.03 ^Ab^	4.39 ± 0.02 ^Bb^
15th day	Decomposed	Decomposed	Decomposed	4.53 ± 0.03 ^Aa^

Means carrying a different superscript capital, with a small letter on the same row, in columns, respectively, are significantly different (*p* < 0.05).

**Table 6:** Pattern of the psychrophilic count (log10 cfu/g) in poultry meat treated with different concentrations of olive leaf extract during the chilling storage period at 4 ± 1 °C (mean ± standard deviation “SD”).

Storage Period	Control	Olive Leaf Extract Concentrations		
		0.25%	0.5%	1%
	Mean ± SD	Mean ± SD	Mean ± SD	Mean ± SD
Zero day	4.23 ± 0.10 ^Ac^	4.01 ± 0.11 ^Bd^	3.86 ± 0.15 ^Cd^	3.70 ± 0.19 ^Ce^
3rd day	4.64 ± 0.06 ^Ab^	4.31 ± 0.09 ^Bc^	4.17 ± 0.03 ^Cc^	3.93 ± 0.03 ^Dd^
6th day	5.19 ± 0.18 ^Aa^	4.53 ± 0.03 ^Bb^	4.18 ± 0.06 ^Cc^	3.99 ± 0.04 ^Dd^
9th day	Decomposed	4.62 ± 0.02 ^Aa^	4.45 ± 0.02 ^Bb^	4.25 ± 0.02 ^Cc^
12th day	Decomposed	Decomposed	4.60 ± 0.03 ^Aa^	4.35 ± 0.05 ^Bb^
15th day	Decomposed	Decomposed	Decomposed	4.53 ± 0.03 ^Aa^

Means carrying a different superscript capital, with a small letter on the same row, in columns, respectively, are significantly different (*p* < 0.05).

**Table 7:** Pattern of the Enterobacteriaceae count (log10 cfu/g) in poultry meat treated with different concentrations of olive leaf extract during the chilling storage period at 4 ± 1 °C (mean ± standard deviation “SD”).

Storage Period	Control	Olive Leaf Extract Concentrations		
		0.25%	0.5%	1%
	Mean ± SD	Mean ± SD	Mean ± SD	Mean ± SD
Zero day	3.02 ± 0.06 ^Ac^	2.92 ± 0.03 ^Ad^	2.82 ± 0.04 ^Cc^	2.56 ± 0.07 ^Dd^
3rd day	3.48 ± 0.07 ^Ab^	3.02 ± 0.06 ^Bc^	2.92 ± 0.02 ^Cc^	2.72 ± 0.04 ^Dd^
6th day	3.81 ± 0.04 ^Aa^	3.21 ± 0.08 ^Bb^	2.96 ± 0.03 ^Cb^	2.86 ± 0.03 ^Dc^
9th day	Decomposed	3.37 ± 0.07 ^Aa^	3.17 ± 0.09 ^Bab^	2.90 ± 0.05 ^Cb^
12th day	Decomposed	Decomposed	3.83 ± 0.6 ^Aa^	3.11 ± 0.13 ^Bab^
15th day	Decomposed	Decomposed	Decomposed	3.31 ± 0.03 ^Aa^

Means carrying a different superscript capital, with a small letter on the same row, in columns, respectively, are significantly different (*p* < 0.05).

**Table 8:** Pattern of the staphylococcal count (log10 cfu/g) in poultry meat treated with different concentrations of olive leaf extract during the chilling storage period at 4 ± 1 °C (mean ± standard deviation “SD”).

Storage Period	Control	Olive Leaf Extract Concentrations		
		0.25%	0.5%	1%
	Mean ± SD	Mean ± SD	Mean ± SD	Mean ± SD
Zero day	4.34 ± 0.08 ^Ac^	4.21 ± 0.01 ^Bd^	4.07 ± 0.07 ^Cd^	3.76 ± 0.11 ^Dd^
3rd day	4.88 ± 0.06 ^Ab^	4.39 ± 0.05 ^Bc^	4.18 ± 0.09 ^Cc^	3.88 ± 0.03 ^Dc^
6th day	5.19 ± 0.04 ^Aa^	4.48 ± 0.03 ^Bb^	4.36 ± 0.02 ^Cb^	3.98 ± 0.03 ^Dc^
9th day	Decomposed	4.67 ± 0.02 ^Aa^	4.38 ± 0.06 ^Bb^	4.01 ± 0.10 ^Cc^
12th day	Decomposed	Decomposed	4.63 ± 0.08 ^Aa^	4.19 ± 0.11 ^Bb^
15th day	Decomposed	Decomposed	Decomposed	4.39 ± 0.03 ^Aa^

Means carrying a different superscript capital, with a small letter on the same row, in columns, respectively, are significantly different (*p* < 0.05).

**Table 9:** The pattern of mold and yeast count (log10 cfu/g) in poultry meat treated with different concentrations of olive leaf extract during the chilling storage period at 4 ± 1 °C (mean ± standard deviation “SD”).

Storage Period	Control	Olive Leaf Extract Concentrations		
		0.25%	0.5%	1%
	Mean ± SD	Mean ± SD	Mean ± SD	Mean ± SD
Zero day	3.77 ± 0.07 ^Aa^	3.66 ± 0.05 ^Ab^	3.51 ± 0.07 ^Bc^	3.10 ± 0.17 ^Bd^
3rd day	4.42 ± 0.13 ^Aa^	3.80 ± 0.04 ^Ab^	3.46 ± 0.15 ^Ab^	3.01 ± 0.17 ^Ac^
6th day	4.72 ± 0.10 ^Aa^	3.96 ± 0.03 ^Ab^	3.77 ± 0.07 ^Ab^	3.46 ± 0.15 ^Ac^
9th day	Decomposed	4.13 ± 0.04 ^Aa^	3.92 ± 0.13 ^Aa^	3.66 ± 0.05 ^Bb^
12th day	Decomposed	Decomposed	4.06 ± 0.06 ^Aa^	3.80 ± 0.04 ^Aa^
15th day	Decomposed	Decomposed	Decomposed	3.93 ±0.03 ^a^

Means carrying a different superscript capital, with a small letter on the same row, in columns, respectively, are significantly different (*p* < 0.05).
